# Editorial: Therapeutic ultrasound in cardiovascular disease

**DOI:** 10.3389/fcvm.2024.1428155

**Published:** 2024-06-03

**Authors:** Guillaume Goudot, Babak Nazer

**Affiliations:** ^1^ Université Paris Cité, INSERM U970 PARCC, Paris, France; ^2^ Vascular Medicine Department, Georges Pompidou European Hospital, APHP, Paris, France; ^3^ Division of Cardiology, University of Washington, Seattle, WA, United States

**Keywords:** therapeutic ultrasound, cavitation, high-intensity focused ultrasound, cardiovascular disease, theranostic application

**Editorial on the Research Topic** Therapeutic ultrasound in cardiovascular disease

“Therapeutic ultrasound” technologies use thermal- and/or pressure-based ultrasound properties to achieve different biological effects and target a growing number of clinical applications. This Research Topic aimed to gather the latest research on cardiovascular applications of therapeutic ultrasound, including all acoustic mechanisms and disease processes. The four manuscripts included in this Research Topic illustrate how acoustic physics can be harnessed to modulate cardiovascular biology and pathology, ultimately developing new therapeutic ultrasound devices and medical applications.

The use of ultrasound as a therapeutic tool is nothing new: the first ultrasound treatment dates back to 1942, when John Lynn experimented with the properties of focused ultrasound on the liver (1), and actually pre-dates diagnostic ultrasound imaging, which emerged in the 1950s. Since then, ultrasound has undergone uninterrupted development. In recent years, however, we have seen the emergence of numerous devices, differing in their presentation (extracorporeal devices, endovascular catheters, etc.), and clinical indications: atherosclerosis, vascular calcifications, arterial and venous thrombosis are just a few examples. Depending on its frequency, intensity, and delivery mode, ultrasound can have very different biological effects: thermal effects, direct mechanical effects, or cavitation induction. In addition, ultrasound can be coupled with the injection of microbubbles carrying active substances, and these microbubbles can be destroyed by ultrasound for localized drug or gene delivery.

In cardiovascular pathology, the endovascular treatment based on lithotripsy for calcified arterial stenosis (2) and using pulsed-cavitation ultrasound with an external device to treat calcified aortic valve (3) have recently brought ultrasound to the forefront. Furthermore, combining therapy with precision ultrasound imaging and the miniaturization of transducers means that ultrasound can now be delivered in a highly localized manner with few systemic side effects.

In this topic, Yin and Jiang review the use of microvesicles for targeted treatment of the myocardium. Among current developments is ultrasound-targeted microbubble destruction, enabling specifically targeted drug delivery to the myocardium. One of the major advantages of using ultrasound in therapeutics is its ability to couple with ultrasonic imaging simply since it uses the same physical modality, enabling a Theranostics approach by coupling diagnostic and therapeutic ultrasound. In cardiac imaging, ultrasound remains the technique with the best temporal resolution, allowing us to follow the movements of the left ventricle throughout the cardiac cycle.

A second application on the left ventricle is developed by Zhan et al. in their review of the literature on the value of contrast-enhanced ultrasound in evaluating and managing coronary microvascular dysfunction. In addition to the advantages of microbubbles' injection to microvascular imaging, research on the therapeutic use of ultrasound to improve coronary microvascular flow is also highlighted, such as using glycoprotein IIb/IIIa-targeted microbubbles (4) or sonothrombolysis (5).

Curini and Pesce reviewed the literature concerning a shock wave approach to managing calcified aortic valves. They highlight the various therapeutic possibilities, with endovascular or external options, for use alone or in conjunction with aortic valve replacement.

In addition to cardiac pathology, there are numerous vascular applications. These include the clinical development of endovascular lithotripsy. There are also developments in the field of sonothrombolysis. As an example, Bader et al. proposed in this topic the evaluation of histotripsy (an ultrasound cavitation-based approach to destroying tissue microscopically) as an approach to thrombolysis. The *ex vivo* evaluation shows the value of coupling therapeutic ultrasound, in this case, using cavitation by a focused transducer with pharmacological thrombolysis to achieve a synergistic effect on thrombus destruction.

The articles included in this Research Topic provide an idea of the wide range of potential applications for therapeutic ultrasound in cardiovascular disease. We are optimistic that these cardiovascular therapeutic ultrasound technologies and others will soon pass scientific and regulatory hurdles and translate to clinical use.
